# Impact of Flash Glucose Monitoring in Adults with Inherited Metabolic Disorders at Risk of Hypoglycemia

**DOI:** 10.3390/nu17020222

**Published:** 2025-01-09

**Authors:** Sandra Amuedo, Elena Dios-Fuentes, Rosa Benítez-Ávila, Pablo Remón-Ruiz, Alfonso Soto-Moreno, Eva Venegas-Moreno

**Affiliations:** Unidad de Gestión Clínica de Endocrinología y Nutrición, Instituto de Biomedicina de Sevilla (IBiS), Hospital Universitario Virgen del Rocío/CSIC/Universidad de Sevilla, Avda. Manuel Siurot s/n, 41013 Seville, Spain; mariae.dios.sspa@juntadeandalucia.es (E.D.-F.); r.beniteza@hotmail.com (R.B.-Á.); pjremonruiz@gmail.com (P.R.-R.); alfonsom.soto.sspa@juntadeandalucia.es (A.S.-M.); evam.venegas.sspa@juntadeandalucia.es (E.V.-M.)

**Keywords:** continuous glucose monitoring, inherited metabolic disorders, hypoglycemia

## Abstract

Background: This study addresses hypoglycemia in adults with inherited metabolic disorders (IMDs), highlighting the importance of intermittently scanned continuous glucose monitoring (isCGM). Despite the elevated risk of hypoglycemia in an important group of these diseases, the use of isCGM remains uncommon and there is limited evidence supporting its effectiveness. Methods: A longitudinal quasi-experimental study was performed in 18 adults with IMDs, evaluating the use of isCGM for 2 months. Time in hypoglycemia (TBR), hyperglycemia (TAR), and time in range (TIR) were monitored, in addition to symptomatic and asymptomatic hypoglycemic events. Follow-up visits were performed at 7 days, 14 days, and 2 months. Results: TBR < 70 mg/dL was significantly reduced from 1.5% at baseline to 0% at 2 months. A decrease in the number and duration of hypoglycemic events was also observed. In some IMD subgroups, isCGM enabled detection of asymptomatic hypoglycemia and adjustment to dietary management, improving glycemic control. Conclusions: isCGM is effective in detecting and reducing hypoglycemia in adults with IMDs, optimizing nutritional therapy, and improving the quality of life of patients and their families.

## 1. Introduction

Hypoglycemia in both children and adults is part of the differential diagnosis of inherited metabolic disorders (IMDs). These disorders represent a complex and heterogeneous group of rare genetic diseases, most of which are inherited in an autosomal recessive manner. They are caused by the absence or hypofunction of an enzyme or its cofactor, leading to the accumulation of substrates or a deficiency of specific metabolites, affecting carbohydrate, fatty acid, and protein metabolic pathways. In recent years, the prognosis of these disorders has improved due to early diagnosis and advances in targeted therapies [1,2].

Dietary treatment is a fundamental pillar in the management of certain groups of these diseases, with the main objective of preventing episodes of hypoglycemia, facilitating metabolic control, and avoiding situations of decompensation. During prolonged fasting or increased catabolism, the body uses compensatory mechanisms, such as glycogenolysis, gluconeogenesis or lipolysis, regulated by enzymes, to obtain the glucose required. However, in certain IMDs, the deficiency or dysfunction of any of these enzymes responsible for maintaining normoglycemia puts patients at increased risk of hypoglycemic events. These hypoglycemic events can be ketotic or non-ketotic, persistent and/or recurrent, and can be fasting or postprandial. They occur asymptomatically in up to half of the cases, and episodes of severe hypoglycemia have been documented [3,4].

The time of onset of hypoglycemia may point to different groups of IMDs. Postprandial hypoglycemia may be an indicator of hereditary fructose intolerance (HFI). On the other hand, fasting hypoglycemia may be associated with glycogen storage diseases (GSDs) type 0, I, III, IV, VI, and IX, which affect hepatic glycogen deposition or release, also known as hepatic glycogenosis; disorders of gluconeogenesis such as fructose-1,6-bisphosphatase deficiency, pyruvate carboxylase deficiency or phosphoenolpyruvate carboxykinase deficiency [5]; fatty acid β-oxidation disorders (FAODs), the most frequent being medium-chain acyl-CoA dehydrogenase (MCAD) deficiency [6,7]; or disorders of ketogenesis, such as 3-hydroxy-3-methylglutaryl-CoA (HMG-CoA) lyase enzyme deficiency. Other metabolopathies, such as glucose transporter type 1 or 2 (GLUT1 and GLUT2) deficiencies, glutamate dehydrogenase 1 (GLUD1) enzyme deficiency, disorders affecting amino acid metabolism such as organic acidemias or other aminoacidopathies, or deficiencies in succinyl-CoA transferase (SCOT) or beta-ketothiolase (BKT) enzymes, responsible for ketolysis defects, also affect glucose homeostasis and increase the risk of hypoglycemia.

The diet should be structured and based on regular intake of slowly absorbed complex carbohydrates to avoid hypoglycemia and achieve good metabolic control. However, it is essential that these diets be personalized to prevent overtreatment and excessive caloric intake, which could lead to obesity, development of type 2 diabetes (T2D) and metabolic syndrome, increasing the risk of atherosclerosis and premature cardiovascular disease [8,9,10,11,12,13,14,15]. For type I GSD, increased frequency and duration of hypoglycemia has been shown to be associated with long-term complications, such as the development of hepatic adenomas [16]. Nevertheless, achieving this control can be challenging, as the fear of hypoglycemia is often present, leading patients to a higher intake than needed.

In children, certain situations necessitate the use of continuous enteral nutrition, particularly overnight, by means of a nasogastric tube, to prevent hypoglycemia. In adults, because fasting tolerance improves with age, the administration of raw corn starch before bedtime, or its modified version, Glycosade^®^, helps to maintain optimal glucose levels [17].

When experiencing symptoms indicative of hypoglycemia, patients are instructed to perform capillary blood glucose self-monitoring. Due to the risk of hypoglycemia, which can be life-threatening, real-time continuous glucose monitoring (rtCGM) or intermittent scanning glucose monitoring (isCGM) systems, also called flash monitoring, offer a promising alternative for glucose monitoring in this population. Currently, the use of these systems is widely standardized in the care of people with diabetes mellitus, with demonstrated benefits in glycemic control and in reducing the risk of hypoglycemia, both in children and adults with type 1 diabetes mellitus [18,19] and in adults with T2D [20].

An increasing number of studies, especially in hepatic GSD, show the usefulness of rtCGM in children and adults, reducing time in hypoglycemia and supporting the optimization of dietary management in these patients [17,21,22,23,24,25,26,27,28,29,30]. In fact, the accuracy of rtCGM has been proven even with levels < 70 mg/dL, favoring its use in clinical decision-making [25]. However, only one clinical case report exists of an adult woman with GSD Ia and secondary diabetes in which isCGM was used to monitor glucose levels and to detect periods of hyperglycemia, allowing improved glycemic control [15].

Despite the high susceptibility of these patients to hypoglycemic events, the use of isCGM remains very limited. This study aims to evaluate the effectiveness and safety of the use of isCGM in different types of IMDs with increased risk of hypoglycemia, as well as to examine the quality of dietary treatment in real-world clinical practice.

## 2. Materials and Methods

### 2.1. Study Design

A prospective longitudinal quasi-experimental study, with a 2-month follow-up, was conducted in a cohort of adult patients with IMDs at increased risk of hypoglycemia, who started using the isCGM system (Freestyle Libre 2 system, Abbot Diabetes Care, Witney, UK).

An in-person training session was conducted on the use of the Freestyle Libre 2 isCGM system, which was placed on the upper arm of each patient. Low glucose alarms were triggered with a target of 70 mg/dL for all participants. Virtual follow-up visits were scheduled at 7 and 14 days, and at 2 months after initiating isCGM use, with interventions by the medical team.

The study protocol followed the Declaration of Helsinki. All participants were informed about the study and signed a consent form.

### 2.2. Study Population

Eighteen adult subjects with IMDs who had experienced episodes of hypoglycemia were selected and started on isCGM (Freestyle Libre 2) for a period of 2 months. All patients had a confirmed diagnosis of IMDs through specific genetic testing.

Inclusion criteria were age > 18 years and any IMDs associated with hypoglycemia. Exclusion criteria included pregnancy and/or presence of intercurrent illness the week prior to the start of the study, defined as fever > 38 °C, decreased intake, vomiting, and diarrhea. These conditions can significantly increase catabolism, which is particularly relevant in patients with IMDs. In these cases, compensatory mechanisms may not be sufficient to meet the increased glucose demand, thereby increasing the risk of hypoglycemia, even beyond the risk present under stable conditions, and potentially introducing bias into the study results.

All patients were treated at the Inborn Errors of Metabolism Unit, pertaining to the Clinical Management Unit of Endocrinology and Nutrition of the Virgen del Rocío University Hospital, which has a multidisciplinary team composed of endocrinologists and a dietician-nutritionist specializing in IMDs.

### 2.3. Data Collection

Glycemic outcomes were compared at baseline and after 2 months of isCGM use. Data collected and evaluated included: time in range (TIR) elapsed in the glucose range 70–180 mg/dL, time in target range (TIT 70–140 mg/dL), time below range (TBR) < 70 and <54 mg/dL, time above range (TAR) > 140, >180 and >250 mg/dL, mean glucose levels, glucose management indicator (GMI), glycemic variability measured by coefficient of variation (CV), sensor usage, and number of scans per day. Baseline data and data obtained at 2 months were downloaded using the Libreview platform, analyzing the download coming from the last 2 weeks of each study period. Glycated hemoglobin (HbA1c) was measured at baseline using methods certified by the National Glycohemoglobin Standardization Program.

### 2.4. Outcomes

The primary objective was to evaluate the presence of hypoglycemia and the change in the percentage of time in hypoglycemia (<70 and <54 mg/dL, and total) at 1 week, 2 weeks, and 2 months of isCGM use, as well as to examine the safety of these systems, as determined by the quantification of acute decompensations requiring health care (episodes of severe hypoglycemia and/or need for hospitalization). Secondary objectives included analysis of the number of hypoglycemic events and their average duration, as well as examining the time of onset of hypoglycemia, the relationship between these events and specific situations, and whether they were symptomatic or asymptomatic, at 1 week, 2 weeks, and 2 months of isCGM use. Changes in TBR, low glucose events and average duration were also described by IMD subgroups, taking into account the modifications made to the participants’ diets.

### 2.5. Statistical Analysis

Results are presented as mean ± SD or median (interquartile range [IQR]) as appropriate to the distribution of the data. The frequency of qualitative variables is expressed as *n* (%). For continuous variables, a Shapiro–Wilk test was used to determine normality. Comparison of continuous variables was carried out using a Friedman’s test for non-normal distributions and a repeated measures ANOVA for normal distributions. A value of *p* < 0.05 was considered statistically significant. Statistical analyses were performed with SPSS version 25.0 software (SPSS Inc., Chicago, IL, USA).

## 3. Results

The study population consisted of 18 isCGM users with IMDs, whose baseline characteristics are shown in Table 1. The mean age was 30.5 ± 12.5 years, with 61.1% men and 100% of Caucasian ethnicity. The mean body mass index (BMI) was 28.1 ± 5.3 kg/m^2^. At baseline, the mean HbA1c was 5.5 ± 0.6%.

Of the total sample, 50% (nine) had FAODs, distributed as follows: four cases of MCAD, one with long-chain acyl-CoA dehydrogenase (LCAD) deficiency, one with carnitine-acylcarnitine translocase (CACT) deficiency, two with carnitine palmitoyltransferase II (CPT II) deficiency, and one with multiple acyl-CoA dehydrogenase (MADD) deficiency.

A total of 27.8% (five) corresponded to GSD. Of these, four cases had liver involvement: two were type IX, one type Ib, and one type IIIa. The remaining case was type V or McArdle’s disease, which affects the muscles.

The remaining IMDs included two cases (11.2%) of glutamate dehydrogenase deficiency (GLUD-1), one case (5.5%) of methylmalonic acidemia (MMA), and one case (5.5%) of HFI.

Before the start of the study and the use of isCGM, all patients followed a diet with regular and controlled carbohydrate intake, in addition to the specific dietary recommendations for each IMD subgroup. As shown in Table 2, many patients consumed raw corn starch (Maizena) in varying amounts before bedtime, with the aim of preventing nocturnal hypoglycemia.

Regarding previous history of hypoglycemia, 50% of the total sample had a history of symptomatic hypoglycemia during the course of their disease, of which 11.1% were severe and 33.3% occurred during the nocturnal period. Table 2 details the characteristics of hypoglycemia by IMD subgroups.

### Hypoglycemia and Safety

Of the entire sample, eight patients (44.4%) had a glucose meter, although they did not regularly monitor their capillary blood glucose levels, either before bedtime or when experiencing symptoms of hypoglycemia.

The glycemic results for the entire cohort are presented in Table 3.

TBR < 70 and 54 mg/dL decreased in the total sample from 1.5% (0–3) at baseline to 0% (0–1) at 2 months (*p* = 0.005), and from 0% (0–2) to 0% (0–0) (*p* = 0.035), respectively. A reduction in total TBR was also observed, from 1.5% (0–4) to 0% (0–1) at 2 months (*p* = 0.021). This reduction in time in hypoglycemia was mainly attributed to the distribution and increased consumption of complex carbohydrates, with no significant increase in time in hyperglycemia. TAR > 140 mg/dL was maintained from 2% (1–7) at baseline to 2% (2–11.5) at 2 months (*p* = 0.167). Glycemic variability showed an improvement, with CV decreasing from 17.4% (14–19) at baseline to 16.6% (14–19) at 2 months (*p* = 0.476). TIR 70–180 mg/dL increased from 98% (94–100) at baseline to 99% (98–100) at 2 months (*p* = 0.020). The mean sensor glucose level remained virtually stable from 103 mg/dL ± 10.3 mg/dL at baseline to 105.3 mg/dL ± 9.7 mg/dL at 2 months (*p* = 0.034).

The number of low glucose events was reduced from 1 (0–7) at baseline to 0 (0–3) at 2 months (*p* = 0.040). The mean duration of these events, measured in minutes, decreased from 61.4 ± 62.5 at baseline to 33.8 ± 41.5 at 2 months (*p* = 0.223). At 2 months, the use of isCGM, assessed by the number of daily scans and the percentage of data captured by the sensor, remained at optimal values.

During the course of the study, six (33.3%) of the participants reported symptoms compatible with episodes of hypoglycemia, most of which were confirmed by capillary blood glucose controls, showing levels < 70 mg/dL. A close correlation was observed between glucose values recorded by isCGM and those measured by a glucometer. Symptomatic hypoglycemia occurred most frequently during the first week of isCGM use and decreased during the second week. The participants who experienced symptomatic hypoglycemia were the two patients with GLUD-1 deficiency, one patient with GSD Ib, one with GSD IIIa, and the two patients with GSD IX. The remaining 12 (66.7%) patients reported no symptoms of hypoglycemia.

Regarding the time of onset, in five patients (27.7%) with GSD IIIa and IX and GLUD-1 deficiency, hypoglycemia was related to prolonged fasting, occurring mainly during the overnight period. In another two patients (11.1%) with GSD Ib and IIIa, hypoglycemia was associated with physical exercise. Finally, in two additional patients (11.1%) with GSD Ib and GLUD-1 deficiency, postprandial hypoglycemia was observed.

No episodes of severe hypoglycemia or hospital admissions related to isCGM use were recorded during the study.

Table 4 describes the analysis of hypoglycemia by IMD subgroups.

After analysis by subgroups, no statistically significant differences were found. Patients with GLUD-1 deficiency presented higher total TBR, <70 and <54 mg/dL, as well as a higher number of hypoglycemic events and longer duration of these events. It is noteworthy that, in the FAODs group, patients with CACT, CPTII, MADD, and LCAD deficiency presented asymptomatic hypoglycemic events that were detected by the use of isCGM, most of them related to prolonged fasting. In contrast, patients with MCAD did not present hypoglycemic events identified by isCGM, nor did the patient with MMA. In the GSD subgroup, all types of hepatic GSD presented episodes of hypoglycemia, both symptomatic and asymptomatic, with the exception of GSD type V (muscular).

Changes were made to the dietary patterns of nine patients during the period of isCGM use to assess its effects. These adjustments mainly involved the distribution and increased consumption of whole-grain carbohydrates in patients with GSD type IIIa or IX, as well as an increase in protein intake, reaching at least 25% of the total caloric intake. In patients with GSD type Ib, complex carbohydrates were distributed, avoiding sucrose, fructose, galactose, and sorbitol intake, and UCCS was administered orally every 4–6 h. In those with LCAD and CACT deficiencies, changes consisting of increased and distributed complex carbohydrates were implemented, along with a restriction of saturated fats and medium-chain triglycerides. Finally, patients with GLUD-1 deficiency followed a low-protein diet with a specific carbohydrate distribution at each meal.

Table 5 presents a detailed analysis of hypoglycemia-related parameters derived from the use of isCGM by FAOD and GSD subgroups.

## 4. Case 1

A 45-year-old man, diagnosed with GSD type Ib since birth, with recurrent epileptic seizures associated with severe hypoglycemia requiring admission to the Intensive Care Unit. He presented with his last severe hypoglycemia in 2020, accompanied by a seizure. Prior to isCGM implantation, the patient consumed 20 g of UCCS before bedtime, and another 20 g at 04:00 a.m., in addition to following a regular, fractionated carbohydrate diet. Despite this supplementation, he continued to experience frequent episodes of symptomatic hypoglycemia, especially in the morning and throughout the day.

**Figure 1:**
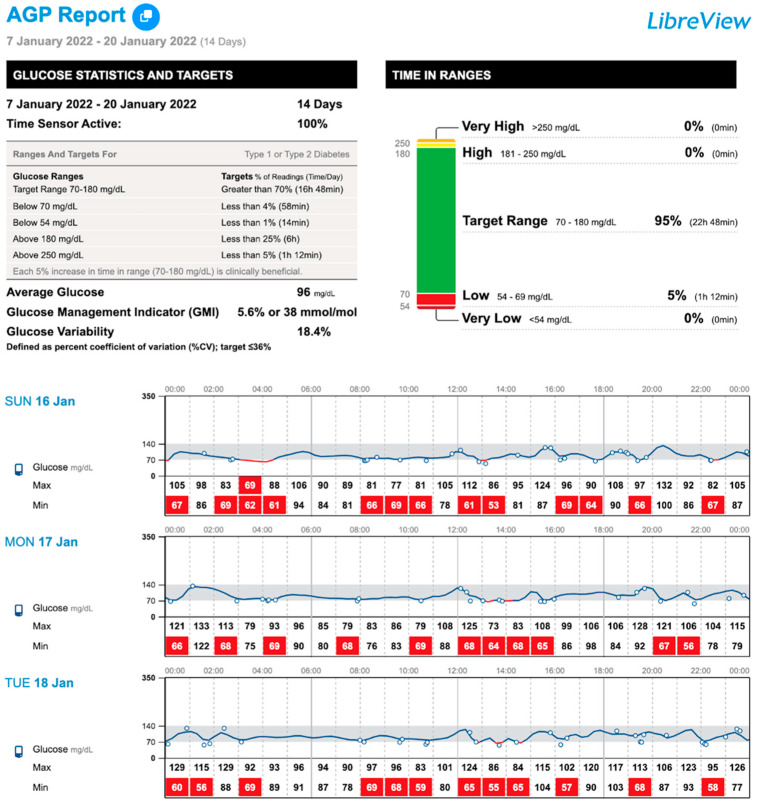
AGP (Ambulatory Glucose Profile) report after 2 weeks of isCGM (Freestyle Libre 2) use in a patient with GSD type Ib and a history of severe hypoglycemia.

With the initiation of isCGM use, as shown in Figure 1, the patient had a TBR < 70 mg/dL of 5%, with symptomatic hypoglycemic events being identified at different times of the day. After analyzing the data obtained from the download, a new supplementation regimen was established with 25 g of UCCS after breakfast, 20 g mid-morning, 20 g after lunch, 20 g after a snack, and the intakes were increased to 30 g after dinner and at 04:00 a.m.

**Figure 2:**
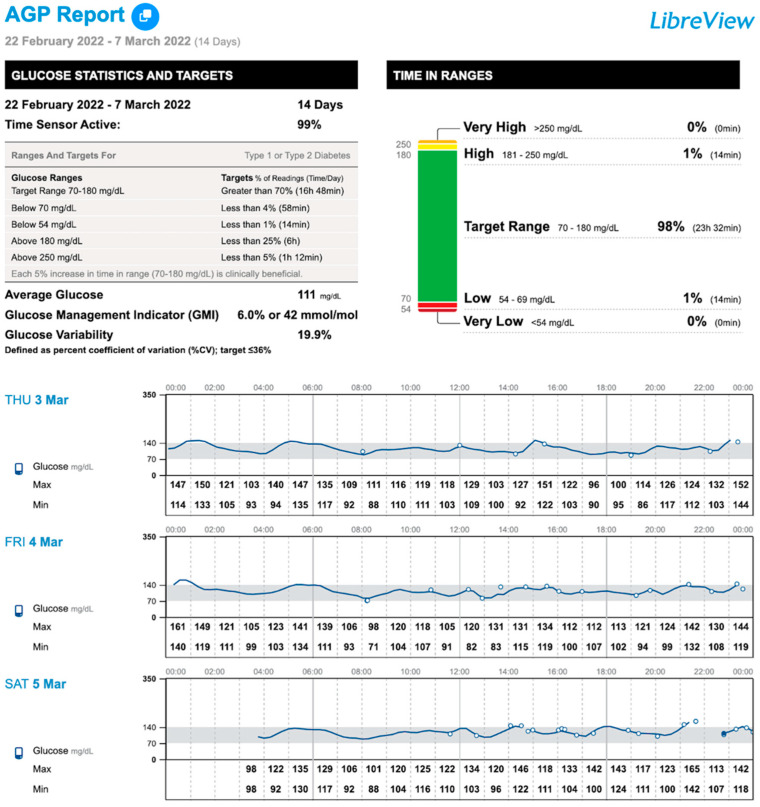
AGP (Ambulatory Glucose Profile) report at 2 months of isCGM (Freestyle Libre 2) use in a patient with GSD Ib and a history of severe hypoglycemia. After dietary modifications, TBR (time below range) < 70 mg/dL decreased.

After these modifications, as seen in Figure 2, TBR < 70 mg/dL decreased from 5% at baseline to 1% at 2 months. It was agreed to maintain a 60% complex carbohydrate diet (excluding fructose, sucrose, and sorbitol, and limiting galactose intake to <2.5 g at breakfast, <5 g at lunch, and <2 g at snack), including UCCS, to avoid possible weight gain, which remained stable, and to prevent secondary hyperinsulinism. Lactate levels were maintained within the normal range. In addition, the time interval between UCCS intakes was extended to every 3–5 h after meals.

## 5. Discussion

In the field of IMDs, despite the increased risk of hypoglycemia in some patients, the use of rtCGM or isCGM remains uncommon. However, an increasing number of studies, particularly in hepatic GSDs, have demonstrated the beneficial effects of these technologies in reducing hypoglycemia rates in both children and adults with IMDs. In this study, following the implementation of the isCGM FreeStyle Libre 2 system in a real-world setting, we demonstrate that adults with different IMDs who are at increased risk of hypoglycemia can safely reduce time in hypoglycemia. Our data show positive effects on time in hypoglycemia (TBR < 70 and <54 mg/dL, as well as total) 2 months after initiation of system use.

To our knowledge, this study is the first to evaluate the effectiveness and safety of the isCGM (FreeStyle Libre 2) system in a real-world clinical setting in adults with IMDs. Previous studies in routine clinical practice have examined the performance of the rtCGM system in children and adults with hepatic GSD, reporting improved detection of asymptomatic hypoglycemic events and improved glycemic control in this population. Herbert et al. reported a TBR < 70 mg/dL between 4% and 8% in 20 users, children and adults, with GSD I, III, and IX after 8.8 ± 5.6 days of rtCGM use, with these being episodes of asymptomatic hypoglycemic [25]. Kasapkara et al. reported an improvement in TBR < 70 mg/dL from 7% to 2.2% after 72 h of rtCGM use and dietary modifications in 16 children with GSD I [24]. In addition, benefits in metabolic parameters and a significant reduction in liver size were reported between 3 and 6 months after use of the system. White et al. identified asymptomatic nocturnal hypoglycemia in a cohort of 23 children and adults with GSD I and GLUT-2 deficiency, enabling the reduction or elimination of nocturnal UCCS intake in those patients who did not have asymptomatic nocturnal hypoglycemia [23].

In our study, TBR < 70 mg/dL in the total sample detected at baseline was 1% (0–3), with a reduction to 0% (0–1) after 2 months of using the isCGM system. Similarly, baseline TBR < 54 mg/dL was 0% (0–3), decreasing to 0% (0–0) at 2 months. This reduction in TBR < 70 and 54 mg/dL is attributed to both the use of low glucose alarms, activated in all patients, as well as increased nocturnal UCCS intake and carbohydrate redistribution. This decrease in time in hypoglycemia did not result in a deterioration of glycemic control, as TIR 70–180 mg/dL increased, while TIR 70–140 mg/dL and >140 mg/dL remained stable after 2 months of isCGM use, also achieving an improvement in glycemic variability. Kaiser et al. reported a duration of up to 2.2 h per day of TBR < 70 mg/dL in a cohort of 14 patients with GSD, of whom 6 (43%) had no symptoms of hypoglycemia [16]. In our study, the average duration of hypoglycemia was reduced from 61 min at baseline to 33 min at 2 months. Similarly, a significant decrease in the number of hypoglycemic events was observed.

Unlike previous studies, this is the first follow-up study with isCGM use of up to 2 months, compared with others that typically used rtCGM for an average of 3 to 13 days. The isCGM system requires the user to actively scan the sensor to retrieve glucose readings, providing snapshots rather than continuous monitoring. In contrast, rtCGM systems continuously transmit glucose data to a receiver or smartphone, offering real-time alerts for hypoglycemia and hyperglycemia [19].

In addition, our cohort consisted exclusively of adult patients, in contrast to other studies in which children predominated. This difference could explain the lower TBR detected at baseline, as adults typically tolerate fasting better than children.

The present work demonstrates that episodes of asymptomatic hypoglycemia are frequent in the daily life of patients with IMDs. Consistent with our results, previous studies have reported the detection of asymptomatic hypoglycemia using rtCGM in patients with GSD [16,23,24,25,29]. Our study, in addition, reports the detection of asymptomatic hypoglycemia events in patients with FAODs, which has not been previously described. Recurrent hypoglycemia causes a defective glucose counter-regulatory response and decreases the perception of hypoglycemia. Detection of asymptomatic hypoglycemia is crucial, as it not only impacts normal development during childhood, but also impaired hypoglycemia awareness puts patients at three to six times greater risk of future severe hypoglycemic events, with increased morbidity, including seizures, coma, and cardiac arrhythmias [31], as well as an increased mortality rate [32], as described in previous studies. Despite the fact that adults have better fasting tolerance, we have identified persistent and recurrent periods of asymptomatic hypoglycemia, especially during the overnight period, in adults through the use of isCGM, which otherwise could not have been detected or corrected. In this regard, both rtCGM [33] and isCGM [18] have been shown to reduce the frequency of severe hypoglycemic events compared with traditional monitoring based on capillary glucose self-testing. These findings support the continued use of isCGM in the adult population with IMDs.

The use of isCGM, in addition to reducing TBR and detecting asymptomatic hypoglycemic events, has proven to be a valuable support to optimize dietary management in these patients. The identification of these events allowed the implementation of appropriate dietary modifications, mainly focused on carbohydrate type, distribution, and increased UCCS intake, especially at night, resulting in improved glycemic control. In our study, virtual follow-up visits were scheduled at 7 and 14 days, and at 2 months after initiation of isCGM use, during which adjustments were made to the dietary and nutritional pattern monitored by the therapeutic team. This demonstrated that isCGM systems are an effective and safe tool to detect asymptomatic hypoglycemia, prevent hypoglycemic events, and support the optimization of nutritional treatment in this population. 

Analysis of the isCGM data by IMD subgroups showed that five patients had no hypoglycemia during the 2-month follow-up. This group included four patients with MCAD and one patient with MMA. Among the patients with MCAD, two had a history of hypoglycemia since childhood, which persisted in one of them until adulthood; this motivated nocturnal supplementation with higher amounts of UCCS and may explain the absence of hypoglycemic events during the use of isCGM. On the other hand, the patient with MMA had no history of hypoglycemia, and no events were identified during the use of isCGM. In one of the patients with CPT II deficiency, asymptomatic hypoglycemic events, predominantly nocturnal, were detected at 2 months. This patient, unlike the others, was not consuming UCCS before bedtime. Similarly, in the patient with MADD, an isolated episode of asymptomatic hypoglycemia was identified, although in this case it was not confirmed by capillary blood glucose control. In the subgroup of patients with GSD, as expected, the patient with muscular GSD did not present hypoglycemia detected by isCGM. The patient with HFI presented asymptomatic hypoglycemic events, mostly nocturnal, reducing TBR < 70 mg/dL from 7% at baseline to 2% at 2 months, after making dietary modifications, excluding foods high in fructose, sucrose, and/or sorbitol, and incorporating a dose of UCCS before bedtime.

Importantly, there is only one clinical case report of an adult woman with GSD Ia and secondary diabetes, in which isCGM was used for 19 days to monitor glucose levels and detect episodes of hyperglycemia, which contributed to improved glycemic control [15]. Our study is therefore the first to use isCGM in a cohort of patients with IMDs during a 2-month follow-up period, assessing both hypoglycemia and dietary treatment adjustment. Moreover, it is the first study to include IMD types other than GSD, such as FAODs, where evidence has so far been limited.

In our study, the assessment of isCGM downloads was performed over a 14-day period, which is the standard in clinical practice and recommended by the International Consensus on Diabetes [34]. In addition, previous studies have shown that 14 days of isCGM data provide an accurate estimate of glucose metrics for a 3-month period [35]. Nevertheless, the recommended glycemic targets, such as TIR, TAR, TBR, and CV, are not yet defined for patients with IMDs.

The main limitations of our study include its nonrandomized design, the absence of a control group, and the small sample size. The inclusion of patients with different types of IMDs could introduce biases in the overall interpretation of the results due to clinical and biochemical heterogeneity. Nonetheless, subanalyses were performed in the different IMD groups and subgroups to mitigate this limitation. Despite these restrictions, the study provides valuable information on the effectiveness and safety of the isCGM system in adults with IMDs susceptible to hypoglycemia in a real-world setting. The findings suggest that the use of isCGM systems could be beneficial as a temporary tool in certain groups of IMDs, depending on age and specific situations, or on an ongoing basis in those groups more prone to persistent and recurrent hypoglycemic events, with the potential to be integrated into the standard of care for the management of these diseases.

## 6. Conclusions

In conclusion, in a real-world clinical setting, the implementation of an isCGM system in adult patients with IMDs allowed the identification of hypoglycemic episodes, including asymptomatic hypoglycemia, achieving a reduction in the rate of hypoglycemic events. In addition, it supported individualized adjustments in dietary treatment to optimize glycemic control and clinical management, improving the quality of life of these patients and their families. To ensure the effective integration of CGM systems into clinical care for these patients, further clinical trials, multicenter studies, and consensus guidelines are essential.

## Figures and Tables

**Table 1:** Baseline characteristics.

Number of Patients	*n* = 18		
Age (years)	30.5 ± 12.5		
Gender (male)—*n* (%)	11 (61.1)		
BMI (kg/m^2^)	28.1 ± 5.3		
HbA1c (%)	5.5 ± 0.6		
IMDs type—*n* (%)			
FAODs	9 (50)	MCAD deficiency (*n* = 4)	
		LCAD deficiency (*n* = 1)	
		CACT deficiency (*n* = 1)	
		CPT II deficiency (*n* = 2)	
		MADD deficiency (*n* = 1)	
GSD	5 (27.8)	Hepatic: 4 (80)	Type Ib (*n* = 1)
			Type IIIa (*n* = 1)
			Type IX (*n* = 2)
		Muscle: 1 (20)	Type V (*n* = 1)
GLUD-1 deficiency	2 (11.2)		
MMA	1 (5.5)		
HFI	1 (5.5)		
History of hypoglycemia—*n* (%)	Symptomatic 9 (50)		
	Nocturnal 6 (33.3)		
	Severe 2 (11.1)		

BMI: body mass index; CACT: carnitine-acylcarnitine translocase; CPT II: carnitine palmitoyltransferase II; FAODs: fatty acid beta-oxidation disorders; GLUD-1: glutamate dehydrogenase; GSD: glycogen storage disease; HbA1c: glycated hemoglobin; HFI: hereditary fructose intolerance; IMDs: inherited metabolic disorders; LCAD: long-chain acyl-CoA dehydrogenase; MADD: multiple acyl-CoA dehydrogenation; MCAD: medium chain acyl-CoA dehydrogenase; MMA: methylmalonic acidemia.

**Table 2:** Dietary regimen and hypoglycemia by IMD subgroups.

IMD Subgroups (*n*)	FAODs (9)					GSD (5)				GLUD-1	MMA	HFI
	MCAD	LCAD	CACT	CPTII	MADD	Ib	IIIa	IX	V			
N	4	1	1	2	1	1	1	2	1	2	1	1
Type of nighttime nutrition												
UCCS	2 (22.2)	1 (11.1)	0	1 (11.1)	0	1 (20)	0	2 (40)	0	1 (50)	0	0
UCCS intake (g/day/person)	40	20	0	20	0	40	0	20	0	20	0	0
History of hypoglycemia												
Childhood	2 (22.2)	0	0	0	0	0	1 (20)	0	0	1 (50)	0	1
Adults	1 (11.1)	0	0	0	1 (11.1)	1 (20)	0	2 (40)	0	2 (100)	0	1
Symptomatic	2 (22.2)	0	0	0	1 (11.1)	1 (20)	0	2 (20)	0	2 (100)	0	1
Nocturnal	2 (22.2)	0	0	0	0	1 (20)	0	1 (20)	0	2 (100)	0	0
Severe	1 (11.1)	0	0	0	0	1 (20)	0	0	0	0	0	0

CACT: carnitine-acylcarnitine translocase; CPT II: carnitine palmitoyltransferase II; FAODs: fatty acid beta-oxidation disorders; GLUD-1: glutamate dehydrogenase; GSD: glycogen storage disease; HFI: hereditary fructose intolerance; IMDs: inherited metabolic disorders; LCAD: long-chain acyl-CoA dehydrogenase; MADD: multiple acyl-CoA dehydrogenation; MCAD: medium chain acyl-CoA dehydrogenase; MMA: methylmalonic acidemia; MMA: methylmalonic acidemia; UCCS: uncooked cornstarch.

**Table 3:** Glycemic outcomes and system usability.

IMDs (*n* = 18)				
	1 Week	2 Weeks	2 Months	*p*
N	18	18	18	
TIR (%, 70–140 mg/dL) ^1^	94 (88–97)	95 (88–97)	94.5 (87–98)	0.214
TIR (%, 70–180 mg/dL) ^1^	98 (94–100) ^a^	98.5 (95–100)	99 (98–100) ^b^	0.020
TAR (%, >140 mg/dL) ^1^	2 (1–7)	2.5 (1–8)	2 (2–11.5)	0.167
TAR (%, >180 mg/dL) ^1^	0 (0–0)	0 (0–0)	0 (0–0.2)	0.751
TAR (%, >250 mg/dL) ^1^	0 (0–0)	0 (0–0)	0 (0–0)	0.368
TBR (%, <70 mg/dL) ^1^	1.5 (0–3) ^a^	1 (0–2)	0 (0–1) ^b^	0.005
TBR (%, <54 mg/dL) ^1^	0 (0–2)	0 (0–0)	0 (0–0)	0.035
TBR total (%) ^1^	1.5 (0–4) ^a^	1 (0–3)	0 (0–1) ^b^	0.021
Low glucose events (*n*) ^1^	1 (0–7) ^a^	1 (0–6)	0 (0–3) ^b^	0.040
Average duration (min) ^2^	61.4 ± 62.5	57 ± 54.4	33.8 ± 41.5	0.223
CV (%) ^1^	17.4 (14–19) ^a^	16.6 (14–19)	16.6 (14–19) ^b^	0.476
Mean glucose (mg/dL) ^2^	103 ± 10.3 ^a^	105.8 ± 11.8	105.3 ± 9.7 ^b^	0.034
GMI (%) ^2^	5.8 ± 0.2	5.8 ± 0.2	5.8 ± 0.2	0.697
Sensor use (%) ^1^	93 (72–99)	93 (86–99)	93 (78–98)	0.129
Scans/day (*n*) ^1^	6 (5–13) ^a^	7 (4–15)	5 (4–12) ^b^	0.021

^1^ Friedman test; ^2^ repeated measures ANOVA; different superscripts letters indicate pairwise differences in follow-up times. Data are presented as median (IQR) in non-normal distributions or as mean ± SD values in normal distributions. CV: coefficient of variation; GMI: glucose management indicator; IMDs: inherited metabolic disorders; TAR: time above range; TBR: time below range; TIR: time in range.

**Table 4:** Hypoglycemia by IMD groups.

IMDs (*n*)	TBR, %			Low Glucose Events (*n*)	Average Duration (Min)
	<70 mg/dL	<54 mg/dL	Total		
FAODs (9)					
1 Week	1.6 ± 3.6	0.4 ± 0.9	2 ± 4.3	2.2 ± 4.1	47.4 ± 81.2
2 Weeks	1.2 ± 2.2	0 ± 0	1.2 ± 2.2	1.9 ± 2.9	42.1 ± 69.4
2 Months	0.3 ± 0.5	0 ± 0	0.3 ± 0.5	1.6 ± 3.6	27.5 ± 33.7
GSD (5)					
1 Week	2.2 ± 0.8	0.4 ± 0.9	2.6 ± 1.5	4.4 ± 4.2	72 ± 25.4
2 Weeks	1.6 ± 0.5	0.2 ± 0.4	1.8 ± 0.8	2.8 ± 1.8	61.6 ± 28
2 Months	0.4 ± 0.5	0 ± 0	0.4 ± 0.5	2.6 ± 2.8	60.8 ± 35
GLUD-1 (2)					
1 Week	6.5 ± 5	0.5 ± 0.7	7 ± 5.6	6 ± 4.2	80 ± 28.3
2 Weeks	5.5 ± 5	2 ± 2.8	7.5 ± 7.7	5.5 ± 5	83.5 ± 0.7
2 Months	2.5 ± 3.5	0 ± 0	2.5 ± 3.5	3 ± 2.8	57 ± 38.2
MMA (1)					
1 Week	0	0	0	1	45
2 Weeks	0	0	0	1	45
2 Months	0	0	0	1	45
HFI (1)					
1 Week	7	1	8	8	151
2 Weeks	5	0	5	10	128
2 Months	2	0	2	3	136

Data are presented as mean ± SD values. FAODs: fatty acid beta-oxidation disorders; GLUD-1: glutamate dehydrogenase; GSD: glycogen storage disease; HFI: hereditary fructose intolerance; IMDs: inherited metabolic disorders; MMA: methylmalonic acidemia; TBR: time below range.

**Table 5:** Hypoglycemia by IMD subgroups.

	FAODs (9)														
	MCAD (4)			LCAD (1)			CACT (1)			CPT II (2)			MADD (1)		
	1 Week	2 Weeks	2 Months	1 Week	2 Weeks	2 Months	1 Week	2 Weeks	2 Months	1 Week	2 Weeks	2 Months	1 Week	2 Weeks	2 Months
TBR (%, <70 mg/dL)	0 ± 0	0 ± 0	0 ± 0	11	5	1	2	5	0	0 ± 0	0 ± 0	0.5 ± 0.7	1	1	1
TBR (%, <54 mg/dL)	0 ± 0	0 ± 0	0 ± 0	2	0	0	2	0	0	0 ± 0	0 ± 0	0 ± 0	0	0	0
TBR total (%)	0 ± 0	0 ± 0	0 ± 0	13	5	1	4	5	0	0 ± 0	0 ± 0	0.5 ± 0.7	1	1	1
Low glucose events (*n*)	0 ± 0	0 ± 0	0 ± 0	11	9	5	3	10	0	0 ± 0	0 ± 0	4.5 ± 4.9	1	1	3
Average duration (min)	0 ± 0	0 ± 0	0 ± 0	151	156	65	216	163	0	0 ± 0	0 ± 0	59 ± 22.6	60	60	65
	GSD (5)														
	Type Ib (1)			Type IIIa (1)			Type IX (2)			Type V (1)					
	1 Week	2 Weeks	2 Months	1 Week	2 Weeks	2 Months	1 Week	2 Weeks	2 Months	1 Week		2 Weeks		2 Months	
TBR (%, <70 mg/dL)	3	1	1	2	2	1	1.5 ± 0.7	1.5 ± 0.7	0 ± 0	0		0		0	
TBR (%, <54 mg/dL)	0	0	0	0	0	0	0 ± 0	0 ± 0	0 ± 0	0		0		0	
TBR total (%)	3	1	1	2	2	1	1.5 ± 0.7	1.5 ± 0.7	0 ± 0	0		0		0	
Low glucose events (*n*)	11	3	2	6	6	5	2 ± 1.4	2.5 ± 2.1	0 ± 0	1		1		0	
Average duration (min)	93	46	40	55	95	50	76 ± 41	78.5 ± 44	0 ± 0	0		0		0	

Data are presented as mean ± SD values. CACT: carnitine-acylcarnitine translocase; CPT II: carnitine palmitoyltransferase II; FAODs: fatty acid beta-oxidation disorders; GSD: glycogen storage disease; IMDs: inherited metabolic disorders; LCAD: long-chain acyl-CoA dehydrogenase; MADD: multiple acyl-CoA dehydrogenation; MCAD: medium chain acyl-CoA dehydrogenase; TBR: time below range.

## Data Availability

The data presented in this study are available on request from the corresponding author as per European legislation on data protection.
